# Supplementary material for: Moderate Aerobic Exercise Training Prevents the Augmented Hepatic Glucocorticoid Response Induced by High-Fat Diet in Mice
Source: Int J Mol Sci. 2020 Oct 14;21(20):7582. doi: 10.3390/ijms21207582 (PMC7590042; doi:10.3390/ijms21207582)
Supplement: Supplementary file 1 [file ijms-21-07582-s001.pdf]

Supplementary Table 1. List of Primers

**Gene**	**Forward primer**	**Reverse Primer**
FKBP5	*5´-CAA TGC TGA GCT TAT GTA CG-3´*	*5´-CTT TTC TTT GGT GTC CAT CTC-3´*
11β-HSD1	*5´-CTC ATA GAC ACA GAA ACA GC-3´*	*5´-TCA AAG GCG ATT TGT CAT AG-3´*
PDK4	*5´-ACA ATC AAG ATT TCT GAC CG-3´*	*5´-TCT CCT TGA AAA TAC TTG GC-3´*
KLF15	*5´-AAC TAG CTT GTT TTG ACA GG -3´*	*5´-AAC ATA TGT ACA CCA GCT TC -3´*
GRα	*5´-GAT GTA TGA AGG CTT TGG TC -3´*	*5´-TGT GCA ACT TTT ATT GGT CTC -3´*
β-Actin	*5´-GAT GTA TGA AGG CTT TGG TC -3´*	*5´-TGT GCA ACT TTT ATT GGT CTC -3´*
YWHAZ	*5´-GAT GTA TGA AGG CTT TGG TC-3´*	*5´-TGT GCA CTT TTA TTG GTC TC -3´*
RPL5	*5´-GAA GAA GAA GAG GTG GAA TC-3´*	*5´-CAG CTT AAC ACA GAA AAA GC -3´*
